# Urinary Tract Infection Caused by Gardnerella vaginalis in a Male Patient With Renal Allograft Transplant

**DOI:** 10.7759/cureus.79847

**Published:** 2025-02-28

**Authors:** Wei-Shin Lu, Michael Kleman, Vaishnavi Aradhyula, Dinkar Kaw, Caleb T Spencer

**Affiliations:** 1 Internal Medicine, The University of Toledo College of Medicine and Life Sciences, Toledo, USA; 2 Nephrology, The University of Toledo College of Medicine and Life Sciences, Toledo, USA

**Keywords:** bacteriuria, gardnerella vaginalis, immunocompromised, renal transplant, urinary tract infection

## Abstract

We present a case of a male patient with a history of renal allograft transplant four years prior to admission who presented with acute kidney injury. There were initial concerns for acute transplant rejection due to elevated creatinine. Urine cultures were positive for *Gardnerella vaginalis*. Renal biopsy results were concerning for chronic pyelonephritis. The patient developed hypertension, fever, and hematuria during the hospital course. He was initially treated with metronidazole, vancomycin, and cefepime. Broad-spectrum antibiotics were quickly discontinued, and the patient improved with metronidazole monotherapy. Patients with a history of renal transplants are often immunocompromised and at risk of infection. The few reported cases primarily involve urinary tract infections. There are scarce reports in the literature of *G. vaginalis* infection in patients with renal allograft transplants, such as in the present case. This case highlights the importance of considering and treating *G. vaginalis* in patients with renal transplants who present with symptoms of urinary tract infection.

## Introduction

*Gardnerella vaginalis* is an anaerobic, Gram-variable bacillus commonly present within the normal vaginal flora in women [1,2]. Clinically, *G. vaginalis* is associated with polymicrobial infections as well as a common cause of bacterial vaginosis in women, a frequently described and easily treated condition [3]. However, reports have also described *G. vaginalis* causing severe infections beyond the genitourinary tract, namely bacteremia, septic arthritis, and pulmonary and brain abscesses [4]. Most of these cases occur in women following surgical instrumentation of the genitourinary tract [4]. However, a much rare presentation of *G. vaginalis* is as an infection in the urinary tract of men, with disseminated infection being even more unlikely. In most men, the presence of *G. vaginalis* in the urethra is insufficient to cause symptoms [1]. Complications may arise once the bacteria invade further to the prostate or bladder, which may be facilitated by urological procedures such as cystoscopy or transurethral resection of the prostate. Furthermore, in patients who are immunocompromised, such as renal transplant patients, the likelihood of dissemination and severe *G. vaginalis* infection becomes more likely. There is scarce literature on *G. vaginalis* bacteremia or bacteriuria in immunocompromised men and no clear consensus on treatment options for male patients with *G. vaginalis* infection. In this study, we seek to highlight the importance of screening and prompt treatment of *G. vaginalis* infection in immunocompromised renal transplant patients.

## Case presentation

A 45-year-old male was admitted for acute kidney injury found on labs from a routine transplant follow-up. His creatinine was 10.6 mg/dL on admission compared to 4.6 mg/dL six months prior to admission. He has a medical history significant for hypertension, type 1 diabetes mellitus, obstructive sleep apnea, and end-stage renal disease secondary to diabetes mellitus, status post renal allograft transplant four years prior to admission from a deceased donor. The patient endorsed that over the past month, he had been more swollen in his bilateral extremities as well as experiencing fatigue, weakness, and decreased energy. Transplant rejection was suspected based on clinical signs and elevated creatinine. Serology labs for the BK virus, Cytomegalovirus (CMV), donor-specific antibody, donor-derived cell-free DNA (Natera Prospera), and urine studies were ordered (Table 1). Labs returned negative except for urine cultures that grew 50,000-100,000 CFU/mL *Gardnerella vaginalis* and confirmed through matrix-assisted laser desorption/ionization-time of flight (MALDI-TOF) mass spectrometry. The patient states that he has not been sexually active with his wife for several months due to erectile dysfunction. He denies dysuria or increased urinary frequency. His diabetes mellitus is well-controlled with insulin.

**Table 1:** Patient laboratory values on admission. eGFR: estimated glomerular filtration rate

Parameters	Patient values	Reference ranges
Urine culture	50,000-100,000 CFU/mL *Gardnerella vaginalis*	-
BK virus	Not detected	-
Cytomegalovirus	Not detected	-
Donor-specific antibody	Not detected	-
Donor-derived cell-free DNA	19 cp/mL	<78 cp/mL: decreased risk for rejection
Blood urea nitrogen	109 mg/dL	7-25 mg/dL
Creatinine	10.64 mg/dL	0.7-1.3 mg/dL
Aspartate aminotransferase	5 U/L	13-39 U/L
Alanine aminotransferase	13 U/L	7-52 U/L
Alkaline phosphatase	47 U/L	34-104 U/L
Bilirubin	0.3 mg/dL	0.3-1 mg/dL
eGFR	5.5 mL/min/1.73 m^2^	>60.0 mL/min/1.73 m^2^

**Figure 1:**
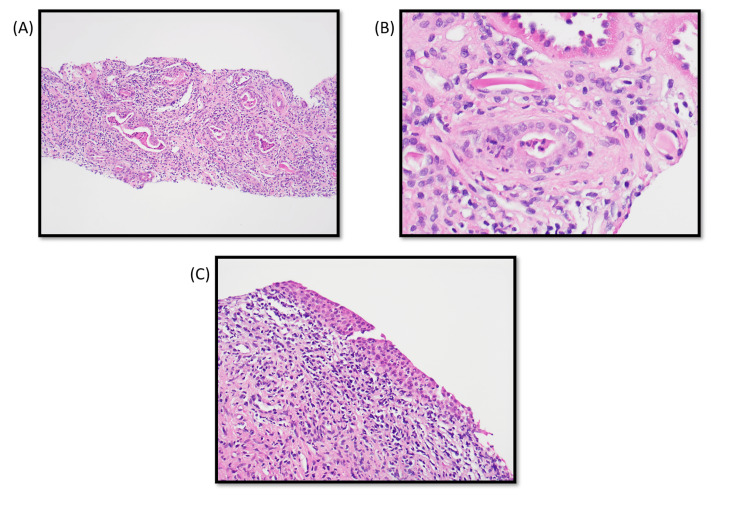
Histopathology images of renal biopsy. (A) Low power view showing interstitial fibrosis with inflammation and dilated tubules containing granular and neutrophilic casts. (B) Neutrophilic tubular cast and neutrophilic tubulitis. (C) Acutely inflamed urothelial lining of a calyx.

A renal biopsy was performed and showed abundant chronic inflammation in the fibrotic areas of the interstitium (Figures 1A-1C). Rare areas also showed neutrophilic interstitial inflammation and tubular neutrophilic casts. Moreover, there was acute inflammation involving a calyceal urothelial lining. The findings were concerning for chronic pyelonephritis.

Following the renal biopsy procedure, the patient developed hypertension, tachycardia, fever, and hematuria. The patient also reported dysuria and difficulty urinating. Labs showed elevated creatinine and an increased absolute neutrophil count (Table 2).

**Table 2:** Patient laboratory values after renal biopsy. eGFR: estimated glomerular filtration rate; HPF: high power field

Variables	Values	Reference ranges
Red blood cells, urine	>100	None seen/HPF
White blood cells, urine	11-20	None seen/HPF
Absolute neutrophil count	26.5 10^3^/µL	1.6-7.6 10^3^/µL
Blood urea nitrogen	100 mg/dL	7-25 mg/dL
Creatinine	11.37 mg/dL	0.7-1.3 mg/dL
Aspartate aminotransferase	6 U/L	13-39 U/L
Alanine aminotransferase	9 U/L	7-52 U/L
Alkaline phosphatase	46 U/L	34-104 U/L
eGFR	5.1 mL/min/1.73 m^2^	>60.0 mL/min/1.73 m^2^
Lactate	1.1 mmol/L	0.5-2.2 mmol/L

Due to suspicion of an infectious process and concern for prostatitis, he received a dose of vancomycin 2000 mg and was started on intravenous (IV) cefepime 1 g every 12 hours as well as metronidazole 500 mg two times a day. The patient's symptomatic episode resolved the next day, and he remained hemodynamically stable. Subsequent blood cultures were negative for infection. Therefore, broad-spectrum antibiotics were discontinued, and the patient continued to improve with metronidazole monotherapy with a total planned duration of four weeks. Repeat urine culture tests were negative. The patient requested to be discharged from the facility. The patient's creatinine level remained high at 12.37 mg/dL (Table 3).

**Table 3:** Lab values of patient prior to discharge. eGFR: estimated glomerular filtration rate

Variables	Values	Reference ranges
Blood urea nitrogen	99 mg/dL	7-25 mg/dL
Creatinine	12.37 mg/dL	0.7-1.3 mg/dL
eGFR	4.6 mL/min/1.73 m^2^	>60.0 mL/min/1.73 m^2^

After consultation for future permanent catheter placement, follow-up for renal function test, follow-up for dialysis, and continued metronidazole therapy for *G. vaginalis*, the patient was discharged home.

## Discussion

*Gardnerella vaginalis* infection is rarely found in men. The prevalence of *G. vaginalis* in both asymptomatic and symptomatic men is debatable, with many studies ranging between 7% and 14% [5]. It is unclear whether colonization is mostly transient or persistent. It is likely that one method for acquiring infection in men is through sexual transmission. Dawson et al. evaluated 430 urethral cultures in men (288 heterosexual, 133 homosexual, and nine bisexual) and found that heterosexual men had a 3.2-fold higher prevalence compared to homosexual men, indicating the likelihood of transmission from female partners [6]. However, there have also been reports of *G. vaginalis* infection in patients who are immunocompromised, have urologic complications such as nephrolithiasis/urolithiasis or stricture, or have undergone urologic procedures such as transurethral prostatectomy and urethral stenting [7-9].

Alfraji et al. describe a man who presented with altered mental status and was subsequently found to have *G. vaginalis* in blood cultures [7]. They were initially treated with ceftriaxone, but after *G. vaginalis* was identified, ceftriaxone was discontinued, and the patient remained stable on metronidazole 500 mg three times a day for 10 days. Lagacé-Wiens et al. describes a previously healthy man who presented with flank pain [10]. Renal stones were discovered, and urine culture was identified and confirmed to reveal *G. vaginalis*. This patient was treated with lithotripsy and improved on ciprofloxacin. Bhatia et al. describes an immunocompromised man due to acquired immunodeficiency syndrome (AIDS) who was found to have *G. vaginalis* on blood cultures [4]. The patient was initially treated with ceftriaxone. However, shortly after *G. vaginalis* identification, management was changed to metronidazole treatment for two weeks.

Commonly, in non-pregnant females, both metronidazole and clindamycin have been shown as effective and are considered first-line antibiotic treatments against symptomatic *G. vaginalis* infection [11]. There are currently no treatment guidelines for *G. vaginalis* infection in males. In addition to treatment methods described previously, other cases of *G. vaginalis* infection were successfully treated with cephalosporins, fluoroquinolones, tetracyclines, and often metronidazole either alone or with combination therapy [10]. In the present case, significant bacteriuria was observed. The patient subsequently developed hypertension, tachycardia, and fever, and there was concern for sepsis. It is possible that the patient’s symptoms were due to a post-biopsy inflammatory response. However, it is worth noting that the patient symptoms immediately improved after prompt treatment with antibiotics. It is also unclear whether the symptoms of sepsis were directly caused by *G. vaginalis*. Notably, blood cultures returned negative for infection. However, no other organism was identified to have likely caused these symptoms on blood or urine culture. It is possible that additional molecular testing would detect an organism that was not identified by standard culture. The patient was initially treated with broad-spectrum antibiotics following this episode, but after no other organisms were identified on blood cultures, antibiotic treatment was narrowed down to only metronidazole. Treatment for this immunocompromised patient was considered based on previous reports of *G. vaginalis* infection in men. Metronidazole was chosen as an appropriate therapy option based on prior case reports and empirical broad-spectrum coverage. The patient's condition continued to improve with metronidazole monotherapy, and subsequent urine and blood cultures were negative for organism growth. This indicates the likely importance of prompt treatment of *G. vaginalis* in immunocompromised men even if asymptomatic to prevent progression to potentially life-threatening complications.

## Conclusions

*Gardnerella vaginalis* in men is rarely reported in clinical practice but is often revealed as an incidental finding or associated with clinical signs of urinary tract infection. Failure to treat infection when discovered in immunocompromised patients can lead to further complications, such as sepsis. As seen in the present case, a patient with a past medical history of renal transplant had findings of *G. vaginalis* bacteriuria. The present case supports the use of metronidazole, which was chosen based on prior case reports as well as being an empirical choice against *G. vaginalis*. While there are no treatment guidelines for *G. vaginalis* bacteriuria in men, our case highlights the importance of prompt treatment of *G. vaginalis* in immunocompromised patients to prevent further complications.
